# Antitumor Activities of Ethyl Acetate Extracts from *Selaginella doederleinii* Hieron *In Vitro* and *In Vivo* and Its Possible Mechanism

**DOI:** 10.1155/2015/865714

**Published:** 2015-03-18

**Authors:** Jia-zhi Wang, Juan Li, Ping Zhao, Wen-tao Ma, Xie-he Feng, Ke-li Chen

**Affiliations:** Hubei University of Traditional Chinese Medicine, Key Laboratory of TCM Resource and TCM Compound Co-Constructed by Hubei Province and Ministry of Education, Wuhan 430065, China

## Abstract

The antitumor activities of ethyl acetate extracts from *Selaginella doederleinii* Hieron (SD extracts) *in vitro* and *in vivo* and its possible mechanism were investigated. HPLC method was developed for chemical analysis. SD extracts were submitted to 3-(4,5-dimethylthiazol-2-yl)-2,5-diphenyl tetrazolium bromide (MTT) assay on different cells, flow cytometry, and RT-PCR analysis using HepG2 cell and antitumor activity *in vivo* using H-22 xenograft tumor mice. Six biflavonoids from SD extracts were submitted to molecular docking assay. The results showed that SD extracts had considerable antitumor activity *in vitro* and *in vivo* without obvious toxicity on normal cells and could induce cell apoptosis. The mechanisms of tumorigenesis and cell apoptosis induced by SD extracts may be associated with decreasing the ratio of bcl-2 and bax mRNA level, activating caspase-3, suppressing survivin, and decreasing the gene expression of COX-2, 5-LOX, FLAP, and 12-LOX mRNA. The main active component in SD extracts is biflavonoids and some exhibited strong interactions with COX-2, 5-LOX, 12-LOX, and 15-LOX. These results offering evidence of possible mechanisms of SD extracts suppress cell proliferation and promote apoptosis and provide the molecular theoretical basis of clinical application of *S. doederleinii* for cancer therapy.

## 1. Introduction

*Selaginella doederleinii* Hieron is a traditional Chinese folk herb which belongs to the family* Selaginellaceae* and is abundant in South and Southwestern China at low altitude [1]. It has been used as folk medicine for the therapy of sore throat, rheumatoid arthritis, and different tumors with a long history, especially for nasopharyngeal carcinoma, choriocarcinoma, lung cancer, and cervical cancer [2–4].

Lian et al. reported that the ethanol extract of* S. doederleinii* can induce mitochondria-related apoptosis in human nasopharyngeal carcinoma CNE cells [5, 6]. Also, researches on the cytotoxic activity against HCT, NCI-H358, K562, and CNE cells of* S. doederleinii* have been reported [1, 6, 7]. Compounds from this herb such as several biflavonoids, lignans, and alkaloids have been reported [7–9]. However, ethyl acetate extracts had stronger antitumor activities than ethanol extract for their abundant biflavonoids, such as amentoflavone, robustaflavone, 2′′,3′′-dihydro-3′,3′′′-biapigenin, 3′,3′′′-binaringenin, heveaflavone, and 7,4′,7′′,4′′′-tetra-O-methyl-amentoflavone [10, 11].

Aberrant arachidonic acid (AA) metabolism is involved in the inflammatory and carcinogenic processes. The effects of biflavonoid mixture from* S. doederleinii* on cyclooxygenase (COX) and lipoxygenase- (LOX-) dependent AA expression in hepatocellular carcinoma were investigated and their effects on cell proliferation and apoptosis were also studied.

## 2. Materials and Methods

### 2.1. Plant Materials

The Chinese herbal* S. doederleinii *was collected from Nanning (Guangxi, China). Identification of specimen was confirmed by Dr. Dingrong Wan, South-Central University for Nationalities (Wuhan, China), and a voucher specimen was deposited in the herbarium of Hubei University of Chinese Medicine, China.

SD extract was obtained by the previously described method [8]. Briefly, the air-dried and powdered samples were extracted twice with petroleum ether and then were filtered. The residues were extracted twice with ethyl acetate and then were filtered. Then the solution was dried using a rotary evaporator, and it was lyophilized and transformed into a power before dissolved in DMSO (dimethyl sulfoxide). The purity of SD extract was about 9.3%.

### 2.2. HPLC Analysis

The detailed method of HPLC analysis could be seen in the literature [11, 12]. Briefly, HPLC analysis was performed on a Dionex HPLC system with P680 Pump, a DiamonsilTM C18 column (250 mm × 5.6 mm, 5 *μ*m), and a UVD 170 U variable wavelength UV-Vis detector. Data were collected and processed using “Chromeleon version 6.0” software. The mobile phase consisted of acetonitrile (A) and water (B). The gradient program was as follows: 25% A in 0–5 min, 25–35% A in 5–12 min, 35–45% A in 12–17 min, 45–50% A in 17–25 min, 50–55% A in 25–40 min, 55–70% A in 40–45 min, 70–100% A in 45–50 min, 100% A in 50–55 min, and 100–25% A in 55–60 min. The flow rate was 1.0 mL/min and column temperature was maintained at 30°C. The injection volume was 10 *μ*L. The detector was set at 330 nm for acquiring chromatograms.

### 2.3. Reagents and Cell Culture

MTT, Trizol, and DMSO were obtained from Sigma (St. Louis, MO, USA). DMEM medium, trypsin, penicillin, and streptomycin were purchased from Gibco (USA). FBS (fetal bovine serum) was bought from Hyclone (USA). All chemicals and reagents were of analytical reagent grade.

HepG2 (hepatocellular carcinoma), Hela (cervical carcinoma), A549 (lung cancer), DU145 (prostatic carcinoma), PC12 (pheochromocytoma), and Vero (African green monkey kidney) cells were obtained from China Center for Type Culture Collection of Wuhan University. The cells were incubated in a humidified atmosphere of 95% air and 5% CO_2_ at 37°C and maintained in DMEM culture medium with 10% FBS plus 100 U/mL streptomycin and 100 U/mL penicillin. The cells were subcultured with 0.25% trypsin when they were 80% confluent. Then, the cells in exponential growth phase were collected for the following experiments.

### 2.4. MTT Assay

Evaluation of antitumor activity* in vitro* was determined with MTT assay which was performed as described before [13]. Briefly, the cells were seeded in 96-well culture plates at a density of 2 × 10^3^ cells per well and then allowed to attach for 24 h before treated with varying concentrations of SD extracts (0, 12.5, 25, 50, 75, 100, and 200 *μ*g/mL) for 72 h. Subsequently, 50 *μ*L MTT of 1 mg/mL was added to each well to react 4 h. The absorbance was determined using the 96-well microplate reader at 570 nm after the formed purple formazan crystals dissolved in 100 *μ*L DMSO. The growth inhibitory ratio was calculated by the following formula: rate of growth inhibition (%) = (1 − OD_treated_/OD_control_) × 100% [14]. The IC_50_ value (concentration of 50% inhibition) was obtained from the dose-response plots of three independent repetitive trials.

### 2.5. Morphology Observation

HepG2 cells were seeded into 24-well plates at a density of 10^4^ cells per well and then exposed to different concentrations of SD extracts for 48 h. The morphological changes of cells were observed and photographed using inverted light microscopy (IX70; Olympus Optical Co., Tokyo, Japan).

### 2.6. Annexin V-FITC/PI Double Staining Assay

Exponentially growing HepG2 cells were placed down in 6-well plate and cultured as above. Then the cells were treated with SD extracts at different concentrations for 24 h. After the drug incubation time, all cells were harvested with trypsin and washed twice with PBS, followed by resuspended in 400 *μ*L Annexin V binding buffer. Then the cells were stained with 5 *μ*L Annexin V-FITC for 15 minutes and 10 *μ*L PI for 5 minutes at 4°C. This assay was performed exactly as the manufacturers' instruction of the Annexin V-FITC cell apoptosis detection kit (BestBio, china). A FACSCalibur flow cytometer was used to detected fluorescence and the percentage of apoptotic cells was calculated by the internal software system of the FACSCalibur. Approximately 10^4^ cells were analyzed for each trail.

### 2.7. RNA Extraction and Real-Time PCR

The mRNA levels of COX-2, FLAP, 5-LOX, 12-LOX, 15-LOX, bcl-2, bax, caspase-3, and survivin were quantified by real-time RT-PCR assays. The *β*-actin was internal reference gene. HepG2 cells were placed down in 6-well plate at a concentration of 5 × 10^5^ cells/mL. After 60% confluency, the cells were exposed to SD extracts at the concentration of IC_50_ for different times (0, 1.5, 3, 6, 9, and 12 h). Then, the cells were harvested to SD extract total RNA using Trizol reagent. A UV spectrophotometer was used to estimate RNA concentration at 260 nm. The purity of RNA was assessed by the ratio of absorbance at 260 and 280 nm (A260/A280 between 1.8 and 2.0). After quantification, 2 *μ*g RNA was used for the synthesis of cDNA in each reverse transcription reaction via TIANScript RT Kit. We conducted PCR to amplify the target genes with reagents and protocols from the SYBR Green PCR Master Mix kit by Invitrogen (Carlsbad, CA, USA). The 20 *μ*L reaction system contained 1 *μ*L generated cDNA template, 1 *μ*L of specific sense primer (Table 1), 1 *μ*L of specific antisense primer, 10 *μ*L 2 × SYBR Green PCR Master Mix, and 7 *μ*L double distilled water. The thermal cycling conditions were 95°C for 15 min, followed by 40 cycles of 95°C for 10 s, 59–63°C (annealing temperature) for 20 s, and 72°C for 30 s and a final incubation at 72°C for 5 min. The relative expression of each gene was normalized to the amount of *β*-actin in the same dosage of cDNA, and the relative quantification method was 2^−ΔΔCT^ method [15]. All RT-PCR reactions for each sample were done in triplicate.

### 2.8. Molecular Docking

We performed a series of molecular docking experiments to estimate the binding affinity of some biflavonoids with COX-2, LOX-5, LOX-12, and LOX-15.

#### 2.8.1. Preparing Protein and Ligands

The three-dimensional structure of biflavonoids was downloaded from PubChem (http://pubchem.ncbi.nlm.nih.gov/) database [16] and refined with the help of Discovery Studio Visualizer (http://accelrys.com/products/discovery-studio/visualization.html). Then, these ligands were converted to MOL2 format using this software. Finally, these mol2 files are optimized by “Prepare Ligands” in Autodock Tools [17] and converted to PDBQT files. The 3D structure of COX-2 (PDBID: 1CX2) [18], LOX-5 (PDBID: 2Q7M) [19], LOX-12 (PDBID: 3RDE) [20], and LOX-15 (PDBID: 1LOX) [21] was obtained from Protein Data Bank, based on their resolution and chemical similarity between bioflavonoids of* S. doederleinii* and the cocrystalled ligands. Protein structure was imported to Autodock Tools. After deleting duplicated chains, removing water and other ligands, and adding hydrogen, the preprocessed structure was assigned with AMBER force field [22] and converted to PDBQT files automatically.

#### 2.8.2. Docking Methodology

Docking procedure can be divided into two parts: grid generation and docking. In the AutoGrid procedure, the target enzyme was embedded on a three-dimensional grid point [17]. The energy of interaction of each atom in the ligand was encountered. In the following phase, Autodock 4.2 was employed in this study. This software uses Lamarckian genetic algorithm (LGA) to perform ligand conformational searching. This algorithm first builds a population of individuals (genes), and then each individual is mutated to acquire a slightly different translation and rotation. The individuals with the low resulting energy after energy minimization process are transferred to the next generation and the process is repeated to obtain the best scored one. For each, binding energy of each pose was calculated using AutoDock 4.2 scoring functions [23].

In the present study, three-dimensional affinity grids of size 60 × 60 × 60 Å with 0.6 Å spacing which centered on the geometric center of cocrystalled ligand were calculated for each of the following atom types: HD, C, A, N, OA, and SA, which represent all possible atom types in a protein and ligand. Additionally, an electrostatic map and a desolvation map were also calculated. Rapid energy evaluation was achieved by precalculating atomic affinity potentials for each atom in the ligand molecule. We have selected important docking parameters for the LGA as follows: population size of 150 individuals, 5 million energy evaluations, maximum of 30000 generations, number of top individuals to automatically survive to next generation of 1, mutation rate of 0.02, crossover rate of 0.8, 10 docking runs, and random initial positions and conformations. The probability of performing local search on an individual in the population was set to 0.06. Top-scored conformation of each ligand was analyzed by AutoDock Tools and Pymol.

### 2.9. Antitumor Activity of SD Extracts* In Vivo*

Kunming mice (male, 20 ± 2 g) were procured from the Center of Experimental Animals in Wuhan University, Wuhan, China. In order to evaluate the antitumor effect of SD extracts* in vivo*, the mice were injected with H-22 (mice hepatoma) cells in the armpit for subcutaneous xenograft tumor models, respectively, except the normal group mice. Then the tumor bearing mice were divided into five groups randomly of which each group has 12 mice. The negative and normal control group mice received only 0.9% normal saline. 5-Fu intraperitoneal injection was taken as positive control. The low-dose, medial-dose, and high-dose drug group were orally administrated with 4, 8, and 16 g/kg/d SD extracts separately. All samples were administrated once every day for 10 days except positive group mice which administrated every other day (10 mg/kg/2d). On the 11th day, all mice were sacrificed, and tumors were excised and weighed for evaluating the tumor growth inhibition. The spleen and thymus were also segregated and weighed to calculate the spleen index and thymus index.

### 2.10. Statistical Analysis

The statistical analysis was evaluated by Student's *t*-test. All data were expressed as mean ± SD. Variance of *P* values obtained was calculated by means of a single-factor ANOVA test. The values of *P* less than 0.05 were considered to be significantly different from each control.

## 3. Results

### 3.1. Fingerprints Analysis

**Figure 1:**
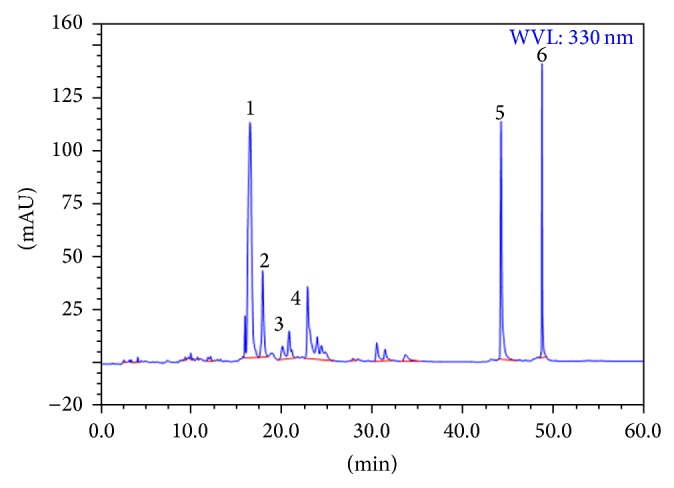
HPLC chromatogram of ethyl acetate extracts. 1: amentoflavone; 2: robustaflavone; 3: 2′′,3′′-dihydro-3′,3′′′-biapigenin; 4: 3′,3′′′-binaringenin; 5: heveaflavone; 6: 7,4′,7′′,4′′′-tetra-O-methylamentoflavone.

HPLC chromatogram was applied for examining biflavonoids from ethyl acetate extracts. As reported in literature [10], the peaks of amentoflavone, robustaflavone, 2′′,3′′-dihydro-3′,3′′′-biapigenin, 3′,3′′′-binaringenin, heveaflavone, and 7,4′,7′′,4′′′-tetra-O-methyl-amentoflavone were marked in Figure 1.

### 3.2. Growth Inhibitory Effects of SD Extract on Different Cells

**Figure 2:**
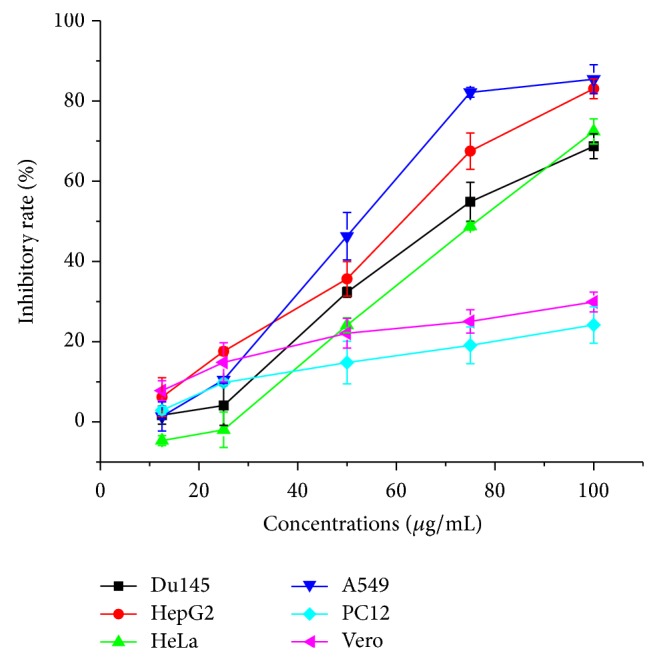
Inhibitory effect of SD extracts on the proliferation of Du145, HepG2, HeLa, A549, PC12, and Vero cells. Cells were treated with different concentrations of SD extracts for 72 h. Values are means ± SD (*n* = 5).

It was widely assumed that MTT has become one of the most widely used methods for measuring cell proliferation and viability. In our study, inhibitory activities against five different tumor cells (DU145, HepG2, HeLa, A549, and PC12) and one normal cell (Vero) were evaluated by MTT method. The data from Table 2 suggested that extracts had definite cytotoxic effect on various cancer cells with a close IC_50_ value except for PC12, which have similar structure and function to the nerve cell and (but) a higher chance of surviving, so it is often used as a cell model to study the nervous system [24]. For this reason, the higher IC_50_ value of SD extracts against PC12 indicated that SD extracts (with little damage to) hurt the central nervous system little and that was also revealed in Figure 2.

As shown in Figure 2, SD extracts caused the tumor cells death in a dose-dependent manner and exhibited apparent cytotoxicity to cancer cells. In addition, for normal cells Vero, SD extracts lead to a growth inhibition rate less than 30%, even at the concentration of 100 *μ*g/mL. The level of cytotoxicity of SD extracts against Vero cells was much lower than cancer cells (Figure 2). The results identified that SD extracts had considerable antitumor activity and low cytotoxicity on normal cells.

### 3.3. Morphological Changes of HepG2 Cells Induced by SD Extracts under Inverted Microscope

**Figure 3:**
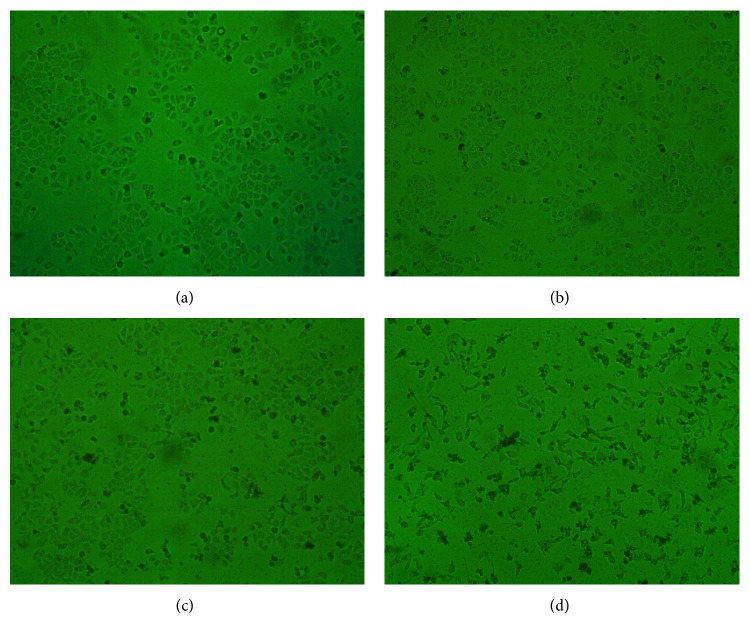
Morphological changes of HepG2 cells induced by different concentration of SD extracts. Changes of cellular morphology were examined at 24 h with 400 magnification. (a) Control; (b) 25 *μ*g/mL; (c) 50 *μ*g/mL; (d) 100 *μ*g/mL.

As shown in Figure 3, morphological changes of HepG2 cells which were treated with SD extracts at different concentrations (25, 50, and 100 *μ*g/mL) were observed as compared with the untreated control cells. Untreated HepG2 cells attached closely on the culture surface in polygon or rotundity with a good refraction and some of them contacted each other to form colonies (Figure 3). However, after treatment with SD extracts, the cells lost their surface morphology significantly and become small and round, made fewer cellular contacts, reduced in size and number. It also can be seen that cell number is depressed obviously. Also these cell morphological changes were in a dose- and time-dependent manner.

### 3.4. Apoptosis Analysis by Flow Cytometry

**Figure 4:**
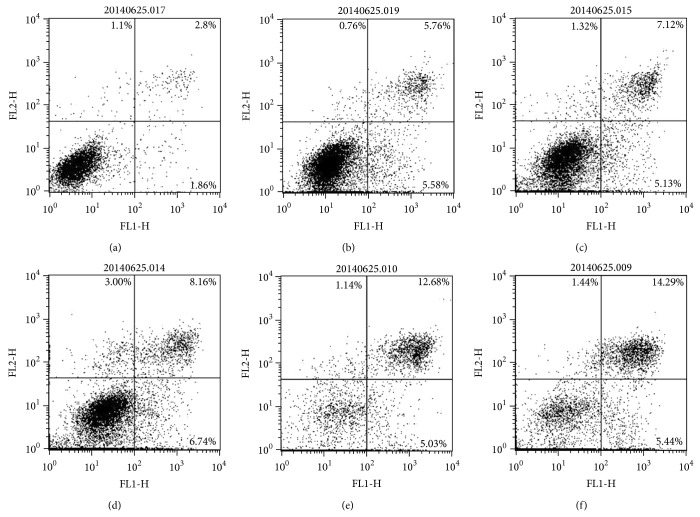
Apoptosis induction by SD extracts in HepG2 cells. Phosphatidylserine translocation of HepG2 cells after SD extracts treatment for 24 h. The population in the region of PI^−^/Annexin V^−^ was considered to be normal cells, the population in the region of PI^−^/Annexin V^+^ was considered to be early apoptotic cells, while that of PI^+^/Annexin V^+^ was considered to be late apoptotic cells. (a) Control; (b) 25 *μ*g/mL; (c) 50 *μ*g/mL; (d) 75 *μ*g/mL; (e) 100 *μ*g/mL; (f) 150 *μ*g/mL.

To determine whether SD extracts-induced cell death was related to apoptosis, Annexin V-FITC/PI staining assay was conducted. Display of phosphatidylserine on the surface of cells, considered as the hallmark of apoptosis in early phase, was determined by PI/Annexin V staining assay. When cells were treated with 25 to 200 *μ*g/mL SD extracts for 24 h, cell populations in late apoptotic phases increased from 5.76% to 14.29%, compared with 2.8% of apoptotic cells in the control (Figure 4).

### 3.5. SD Extracts Changed mRNA Expression of Proinflammatory Factors and Apoptosis-Related Genes

**Figure 5:**
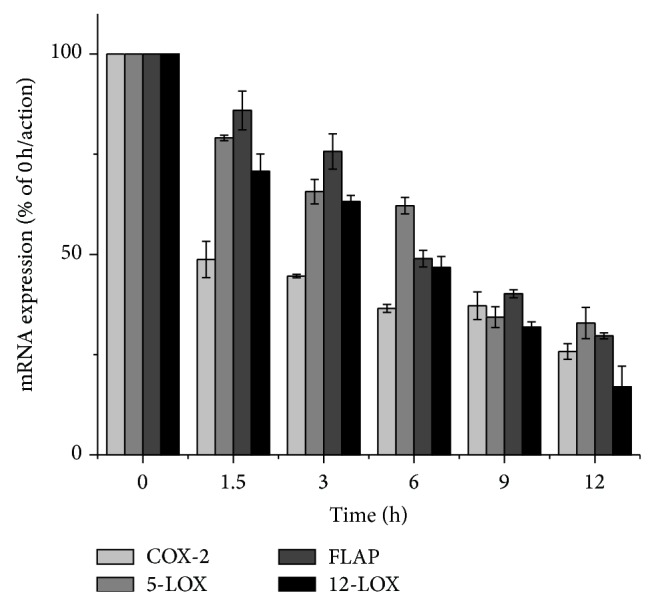
Effects of SD extracts on COX-2, 5-LOX, FLAP, and 12-LOX mRNA expression. HepG2 cells were treated with SD extracts (65.8 *μ*g/mL) for 0, 1.5, 3, 6, 9, and 12 h. COX-2, 5-LOX, FLAP, and 12-LOX mRNA levels were determined by RT-PCR. Values are means ± SD (*n* = 5).

We preformed RT-PCR assay to determine whether SD extracts treatment changed expression of several proinflammatory genes and apoptosis-related genes, which have been verified relevant to cell proliferation, apoptosis, invasion, metastasis, and angiogenesis [25]. As seen in Figure 5, the level of COX-2, 5-LOX, FLAP, and 12-LOX mRNA was markedly decreased by SD extracts in HepG2 cells compared with untreated cells, which implied SD extracts might restrain proinflammatory cytokines production at gene level. In the meanwhile, the mRNA expression of 15-LOX which was generally thought to present tumor inhibition effect increased significantly up to 5.51 times higher than control group (data was not shown). In addition, the expression of COX-2, 5-LOX, FLAP, 12-LOX, and 15-LOX mRNA levels changes regularly with the action time of SD extracts extending in 12 h. The treatment of SD extracts on HepG2 cells for 12 h inhibited COX-2, 5-LOX, FLAP, and 12-LOX mRNA production up to 74.22%, 67.1%, 71.3%, and 83.03%, respectively. These results demonstrated that SD extracts treated for indicated time would resulted in regression of inflammatory in a time-dependent manner, and its effect of inhibiting cell proliferation and promoting cell apoptosis were related with down-regulation of COX-2, 5-LOX, FLAP and 12-LOX and up-regulation of 15-LOX.

**Figure 6:**
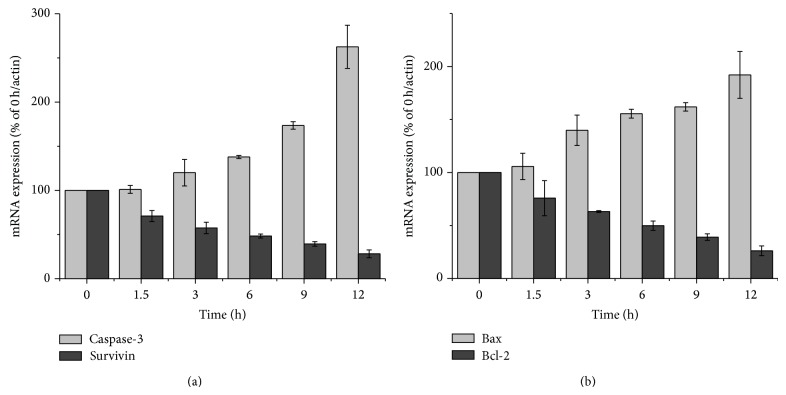
Effects of SD extracts on mRNA expression of apoptosis-related genes. (a) mRNA expression of caspase-3 and survivin genes in HepG2 cells treated with SD extracts (65.8 *μ*g/mL) for 0, 1.5, 3, 6, 9, and 12 h. Values are means ± SD (*n* = 5). (b) mRNA expression of bax and bcl-2 genes in HepG2 cells treated with SD extracts for 0, 1.5, 3, 6, 9, and 12 h. Values are means ± SD (*n* = 5).

In another aspect, bcl-2 and bax are a pair of momentous apoptosis genes, the ratio of bax to bcl-2 determines whether a cell undergoes apoptosis [26], and survivin can prevent and attenuate cell apoptosis markedly [27]. mRNA expression levels of antiapoptotic genes bcl-2 and survivin, proapoptotic gene bax, and caspase-3 were also determined by RT-PCR. Survivin and bcl-2 mRNA expression were decreased, whereas that of bax and caspase-3 were increased in time-dependent manner after SD extracts treatment (Figure 6). As shown in Figure 6, bax and caspase-3 genes were observed to be induced by about 1.92- and 2.62-fold, respectively, whereas bcl-2 and survivin were repressed by about 73.9% and 71.8% after SD extracts treatment compared to untreated cells. These results demonstrated that one of the possible mechanisms of SD extracts induced apoptosis was associated with expression changes of bax, bcl-2, caspase-3, and survivin.

### 3.6. Molecular Docking

**Figure 7:**
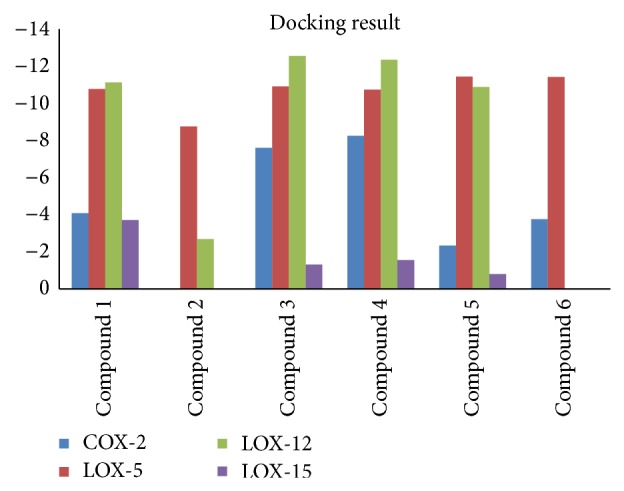
Quantify the interaction between biflavonoids and COX-2, LOX-5, LOX-12, and LOX-15 by AutoDock program. 1: amentoflavone; 2: robustaflavone; 3: 2′′,3′′-dihydro-3′,3′′′-biapigenin; 4: 3′,3′′′-binaringenin; 5: heveaflavone; 6: 7,4′,7′′,4′′′-tetra-O-methylamentoflavone.

**Figure 8:**
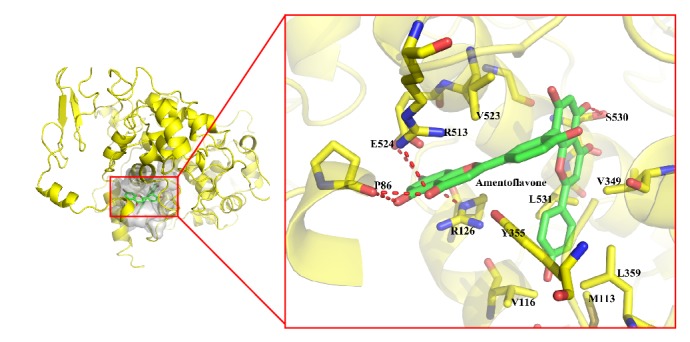
The binding mode analysis of amentoflavone with COX-2. The interactions were analyzed by Pymol.

The docking process was accomplished by AutoDock (version 4.2) and the docking results were quantified by AutoDock 4.2 scoring functions. The docking score for biflavonoids could be seen in Figure 7, 3′,3′′′-binaringenin exhibited strong interactions with COX-2, all compounds showed good interactions with LOX-5, and amentoflavone, 2′′,3′′-dihydro-3′,3′′′-biapigenin, 3′,3′′′-binaringenin, and heveaflavone might have potential better binding ability with LOX-12, while all compounds showed weak interactions with LOX-15. However, robustaflavone exhibited no interactions with COX-2 and LOX-15, and 7,4′,7′′,4′′′-tetra-O-methylamentoflavone exhibited no interactions with LOX-12 and LOX-15. The binding mode analysis of amentoflavone with COX-2 could be seen in Figure 8.

### 3.7. Antitumor Activity* In Vivo*

Due to the good antitumor activities* in vitro* of SD extracts, they were examined for animal studies. As daily observation, no mice dead when treated with SD extracts. The effects of SD extracts on mice transplanted with H-22 were presented in Table 3. The results revealed that SD extracts significantly decreased the tumor weights of H-22 tumor-bearing mice. The inhibitory rates were 23.8%, 39.8%, and 57.7% at the dosages of 4, 8, and 16 g/kg/day, respectively. Furthermore, SD extracts could decrease the spleen and thymus index of the tumor-bearing mice (Table 3).

## 4. Discussion

In the present study, we investigated the anticancer effects of SD extracts on tumor cells and the possible mechanisms. MTT assay proved that SD extracts significantly exhibited antiproliferation activity on various carcinoma cells with a low IC_50_ value and had little damage on Vero and PC12 cells, demonstrating its selective antitumor action to some degree and potential practical valuableness in the therapy for cancer. However, more in-depth research on the detailed mechanisms of SD extracts killing tumor cells rather than normal cells is needed.

Several proinflammatory mediators have been confirmed to play a significant role in inhibition of angiogenesis, apoptosis, proliferation, and metastasis [28]. Both COX and LOX pathway function as a crucial mediator of cell survival and apoptosis [29–31]. COX-2 has been implicated in the growth and progression of a variety of human cancers, 5-LOX and FLAP play a vital role in tumor cells growth related signal transduction and can stimulate oncogene expression, and 12-LOX may be responsible for the adhesion, invasion, and metastasis of cancer cells and also can promote tumor angiogenesis [30]. However, 15-LOX is now considered to be a tumor-inhibiting factor and mainly inhibit carcinoma cells growth. Our RT-PCR results indicated that the mRNA expression of COX-2, 5-LOX, FLAP, and 12-LOX decreased and 15-LOX increased in HepG2 cells after SD extracts treatment. The susceptibility to apoptosis by SD extracts is associated with the level of COX-2 and LOXs in HepG2 cells, which present high COX-2 expression spontaneously. Therefore, SD extracts, as agents which bring the gene expression of COX-2, 5-LOX, FLAP, and 12-LOX mRNA down, should play an inhibitory role in tumorigenesis and metastasis and induce carcinoma cells apoptosis.

The main active component in SD extracts is biflavonoids, and different biflavonoids exhibited interactions with COX-2, 5-LOX, 12-LOX, and 15-LOX in varying degrees. This study just analyzed six biflavonoids, and there were other compounds in SD extracts, and more works are needed.

We also utilized an RT-PCR method to quantitate the expression of both bcl-2, bax, caspase-3, and survivin mRNA expression. The results showed that one of the mechanisms of cell apoptosis induced by SD extracts may decrease the ratio of bcl-2 and bax mRNA level, activate caspase-3, and suppress survivin to promote cell apoptosis.

## 5. Conclusion

In summary, SD extracts had considerable antitumor activity* in vitro *and* in vivo *without obvious toxicity on normal cells and could induce cell apoptosis. The mechanisms of tumorigenesis and carcinoma cell apoptosis induced by SD extracts may be associated with decreasing the ratio of bcl-2 and bax mRNA level, activating caspase-3, suppressing survivin, and decreasing the gene expression of COX-2, 5-LOX, FLAP, and 12-LOX mRNA. The main active component in SD extracts is biflavonoids, and some exhibited strong interactions with COX-2, 5-LOX, 12-LOX, and 15-LOX.

## Figures and Tables

**Table 1:** Primer list.

Gene	Forward primer (5′ to 3′)	Reverse primer (5′ to 3′)
5-LOX	GCCTCCCTGTGCTTTCC	ACCTGGTCGCCCTCGTA
FLAP	GCTGCGTTTGCTGGACTGATGTA	TAGAGGGGAGATGGTGGTGGAGAT
12-LOX	CTTCCCGTGCTACCGCTG	TGGGGTTGGCACCATTGAG
15-LOX	CTGGAGCCTTCCTAACCT	GTGACAAAGTGGCAAACC
COX-2	TATGAGTGTGGGATTTGACCAG	TCAGCATTGTAAGTTGGTGGAC
bcl-2	GTGGAGGAGCTCTTCAGGGA	AGGCACCCAGGGTGATGCAA
bax	GGCCCACCAGCTCTGAGCAGA	GCCACGTGGGCGTCCCAAAGT
caspase-3	ATGGAGAACACTGAAAACTCAGT	TTAGTGATAAAAATAGAGTTCTTTTGT
survivin	ATGGGTGCCCCGACGTTGCCCCCT	TCAATCCATGGCAGCCAGCTGCTCG
*β*-actin	TGACGTGGACATCCGCAAAG	CTGGAAGGTGGACAGCGAGG

**Table 2:** IC_50_ values for SD extracts on the proliferation of different cells (*μ*g/mL).

	DU145	HeLa	A549	HepG2	PC12	Vero
SD extracts	70.5 ± 2.6	76.1 ± 1.9	51.9 ± 1.5	65.8 ± 4.4	>150	>150

Values are means ± SD (*n* = 5).

**Table 3:** Antitumor effects of SD extracts against tumor growth on H-22 tumor-bearing mice.

		Tumor weight (g)	Tumor growth inhibition (%)	Spleen index (mg/10 g)	Thymus index (mg/10 g)
H22	Control	1.46 ± 0.30		5.62 ± 0.83	2.02 ± 0.42
	16 g/kg	0.62 ± 0.27^**^	57.7	4.96 ± 0.72	1.64 ± 0.24^*^
	8 g/kg	0.88 ± 0.28^*^	39.8	5.30 ± 1.02	1.79 ± 0.38
	4 g/kg	1.12 ± 0.40	23.3	5.55 ± 0.95	1.90 ± 0.32
	5-Fu	0.60 ± 0.15^**^	58.9	4.76 ± 0.72^*^	1.42 ± 0.2^**^

Values represent mean ± SE. ^*^ *P* < 0.05 compared to the control group; ^**^ *P*< 0.01 compared to the control group.

## Abbreviations

AA: Aberrant arachidonic acid
IC_50_: Concentration of 50% inhibition
COX: Cyclooxygenase
DMSO: Dimethyl sulfoxide
FBS: Fetal bovine serum
HEp-2: Human laryngeal carcinoma cells
LOX: Lipoxygenase
MTT: 3-(4,5-Dimethylthiazol-2-yl)-2,5-diphenyl tetrazolium bromide
PI: Propidium iodide
SD extracts: Ethyl acetate extract of* Selaginella doederleinii*.
